# By promoting growth and development, castor bean meal biofertilizer improves the yield and quality of Tartary buckwheat and indirectly improves the growth and development of Tartary buckwheat sprouts

**DOI:** 10.3389/fpls.2025.1584608

**Published:** 2025-06-05

**Authors:** Li Mingjing, Hu Xuemei, Luo Rui, Zhang Chunhua, Hu Ruimei, Xue Guibin, Li Guorui, Di Jianjun, Wang Cheng, Gu Xiaohui, Su Zhimin, Li Ruxin, Zhao Yong, Huang Fenglan

**Affiliations:** ^1^ College of Life Science and Food, Inner Mongolia Minzu University, Tongliao, China; ^2^ Tongliao Scientific Research Institute of Agriculture and Animal Husbandry, Tongliao, China; ^3^ Beijing Zhongmin Sunshine Biotechnology Co, Beijing, China; ^4^ College of Life Science, Baicheng Normal University, Baicheng, China; ^5^ Key Laboratory of Castor Breeding of State Ethnic Affairs Commission, Tongliao, China; ^6^ Inner Mongolia Key Laboratory of Castor Breeding and Comprehensive Utilization, Tongliao, China; ^7^ Inner Mongolia Engineering Research Center of Industrial Technology Innovation of Castor, Tongliao, China; ^8^ Inner Mongolia Industrial Engineering Research Center of Universities for Castor, Tongliao, China

**Keywords:** castor bean meal biofertilizer, Tartary buckwheat, Tartary buckwheat sprouts, yield, quality

## Abstract

**Introduction:**

Fertilizer selection and application is closely related to crop yield and quality. Tartary buckwheat is a medicinal and food crops, has a broad space for development. However, the effect of castor bean meal biofertilizer on the growth and development, yield and quality of Tartary buckwheat and Tartary buckwheat buds is not clear. The aim of this study was to elucidate the effect of castor bean meal biofertilizer on Tartary buckwheat yield and quality, and then to elucidate the effect of castor bean meal biofertilizer indirectly on Tartary buckwheat bud yield and quality.

**Methods:**

Tong buckwheat 3 as the test material, in 2 years of field trials, no fertilizer, chemical fertilizer and cow manure as a control, a total of 10 types of fertilizer treatment, respectively, CK (0 kg·ha^-1^), F1, F2, F3 (fertilizer, 225, 300, 375kg·ha^-1^); N1, N2, N3 (cow manure, 7500, 15000, 22500kg·ha^-1^); B1, B2, B3(Castor bean meal biofertilizer, 7500, 15000, 22500kg·ha^-1^).

**Results:**

(1) under different fertilizer treatments, Tartary buckwheat plant height, stem thickness, the number of main stem nodes, the number of main stem branches, leaf area and chlorophyll content; single plant grain weight, thousand grain weight and yield of castor bean meal biofertilizer treatment is good, and in the B2 treatment to reach the maximum value. (2) under different fertilizer treatments, Tartary buckwheat protein, starch, cellulose, fat, flavonoid fractions and bioflavonoids are B2 treatment when the highest content. (3) Different fertilization treatments of Tartary buckwheat cultivated Tartary buckwheat buds bud length, fresh weight and dry weight there are significant differences. Tartary buckwheat buds in the 0-16d free amino acids, soluble sugars, total phenols, vitamin C and bioflavonoids content of Tartary buckwheat seeds in the B2 treatment of Tartary buckwheat cultivated Tartary buckwheat buds of the best indicators.

**Discussion:**

In short, this study provides a new fertilization option to improve Tartary buckwheat yield and quality.

## 1 Introduction

Buckwheat(*Fagopyrum esculentumMoench.*) is an annual dicotyledonous herbaceous plant in the family Polygonaceae and genus *Fagopyrum* (Fan et al., 2021). Buckwheat is widely planted in Asia, Europe, and the Americas and is mainly distributed in China, Russia, France, and Ukraine (Jha et al., 2024). There are three main cultivars of buckwheat in China: common buckwheat, Tartary buckwheat, and sweet buckwheat. Buckwheat planting is distributed mainly in the southwest and northern regions of China. Hure Banner in Tongliao city, Inner Mongolia Autonomous Region, known as the “Hometown of Buckwheat in China”, is mainly planted with the Tartary buckwheat(*Fagopyrum tataricum (L.) Gaertn.*) variety (He et al., 2024). Tartary buckwheat is rich in protein, starch, fat, dietary fiber, minerals, vitamins, and a variety of bioactive components (flavonoids, phenolic acids, and alkaloids) (Sonawane et al., 2024). Tartary buckwheat has hypoglycemic, hypolipidemic, antioxidant, anticancer, and antitumor effects and prevents coronary heart disease (Zhang et al., 2012; Gimenez-Bastida and Zielinski, 2015). Therefore, Tartary buckwheat is considered a medicinal and edible crop and has great development and economic value.

Studies have shown that fertilization is the most effective method for improving crop yield and quality (Wang et al., 2020). The application of an appropriate amount of chemical fertilizer can increase the nutrient content in the soil so that crops can quickly obtain sufficient nutrients for crop growth and development (Wang et al., 2024; Ahmed et al., 2021). However, overfertilization not only leads to soil compaction and acidification, a microbial proportion imbalance, and a decrease in fertilizer use efficiency but also causes imbalances in crop nutrition, hinders internal synthesis and transformation mechanisms, and ultimately affects the yield and quality of crops (Miao et al., 2010; Sparks, 2020). Therefore, coordinating the relationship between the fertilizer application rate and crop yield and quality is important for achieving high and stable crop yields, improving crop quality, and achieving safe production.

At present, the most common fertilizers on the market are divided into three types: chemical fertilizers, organic fertilizers, and bioorganic fertilizers (Bhunia et al., 2021). The extensive use of chemical fertilizers has led to serious incidental problems, such as environmental pollution, chemical resistance of pests, and reduced food safety (Khatri et al., 2023). Owing to the need for sustainable agricultural development, increasing research has been devoted to the study of bioorganic fertilizers that are less harmful to the environment, crops, and food. Castor bean meal (CBM) biofertilizer is a kind of bio-organic fertilizer formed by the fermentation of beneficial bacteria and soil enzymes, Compared with other fertilizers, CBM biofertilizer is not only rich in organic matter but also contains many beneficial microorganisms. These beneficial microorganisms can form symbiotic relationships with soil microorganisms to inhibit the development of harmful bacteria and promote healthy crop growth. In recent years, many scholars have conducted systematic studies on the effects of bioorganic fertilizers on crops. Lin et al. (2024) reported that the application of an appropriate amount of bioorganic fertilizer effectively improved soil fertility and significantly increased yield indicators such as fresh weight, dry weight, and the content of bioactive components in lettuce. Liu et al. (2023) reported that the addition of bioorganic fertilizers such as *Bacillus megaterium*, *Bacillus mucilaginosus*, and *Bacillus subtilis* resulted in the most significant improvement in tea yield and quality, and compared with conventional chemical fertilizers, the qualities of tea polyphenols, amino acids, and caffeine in tea treated with the three bioorganic fertilizers were significantly higher. Some scholars have conducted relevant research on buckwheat. Wan et al. (2023) reported that bioorganic fertilizers improved agronomic traits such as plant height and yield, promoted plant growth and dry matter accumulation, and increased the contents of protein, starch, and bioflavonoids in buckwheat. Tao et al. (2023) reported that the starch content of Tartary buckwheat first increased but then decreased with increasing bioorganic fertilizer application rate. Preliminary studies have shown that castor cake fertilizer significantly improves the root length, plant height, stem diameter, fresh weight, and dry weight of rapeseed. Li et al. (2024) used three types of fertilizers, inorganic fertilizer, organic fertilizer, and castor cake fertilizer, separately during peanut cultivation and reported that, compared with the other two types of fertilizers, castor cake fertilizer not only improved the soil physicochemical environment but also substantially improved peanut agronomic traits and yields, promoted the synthesis of peanut nutrients, and further enhanced the high quality and high yield of peanuts. Suzuki et al. (2021) reported that, unlike the differences in the quality of buckwheat grains harvested under different fertilization treatments, the differences in the growth and development and nutrients of buckwheat sprouts bred from buckwheat treated with different fertilizers were even greater. To date, there are fewer studies on CBM biofertilizer, so it is important to understand the effect of CBM biofertilizer on buckwheat yield and quality for the development and utilization of CBM biofertilizer.

Based on the above studies, the hypotheses of this study are as follows: (i) CBM biofertilizer may improve the yield and quality of buckwheat by regulating the plant height, stem diameter, number of main stem nodes, number of main stem branches, leaf blade area, and chlorophyll content of Tartary buckwheat; (ii) the length, sprout diameter, tap root length, fresh weight, dry weight, and contents of free amino acids, soluble sugars, vitamin C, bioflavonoids, and total phenols of buckwheat sprouts grown from grains harvested under different fertilization treatments may be different. However, relevant studies are lacking. Therefore, a Tartary buckwheat variety named Tongqiao No. 3 was used as the experimental material in this study, and different application rates of chemical fertilizer, cow manure, and CBM biofertilizer were employed to evaluate the effects of CBM biofertilizer on the agronomic traits, yield, and quality of Tartary buckwheat and Tartary buckwheat sprouts. The main purpose of this study was to reveal the effects of CBM biofertilizer on the growth, development, yield, and quality of Tartary buckwheat. The results of this study provide a theoretical basis and technical reference for the high-quality and high-yield cultivation of Tartary buckwheat.

## 2 Materials and methods

### 2.1 Test material and growth

The Tartary buckwheat variety used in the experiments was “Tongqiao No. 3”, which was provided by the Key Laboratory of Castor Breeding of the State Ethnic Affairs Commission. Buckwheat sprouts were cultivated from the Tartary buckwheat seeds grown in this study. The experiment was conducted during the buckwheat growing season (July-October) in 2021–2022 at the experimental field of the Institute of Agricultural and Animal Husbandry Sciences, Tongliao City, Inner Mongolia Autonomous Region (altitude 178 m, 122°32′E, 43°44′N). The soil type was sandy loam soil. The nutrient contents of the plow layer (0–20 cm) were as follows: available nitrogen content, 70.76 mg·kg^-1^; available phosphorus content, 83.10 mg·kg^-1^; available potassium content, 255.91 mg·kg^-1^; organic matter content, 14.76 g·kg^-1^. The soil nutrient content was measured by Nanjing Cavens Testing Technology Co., Ltd.

**Figure 1:**
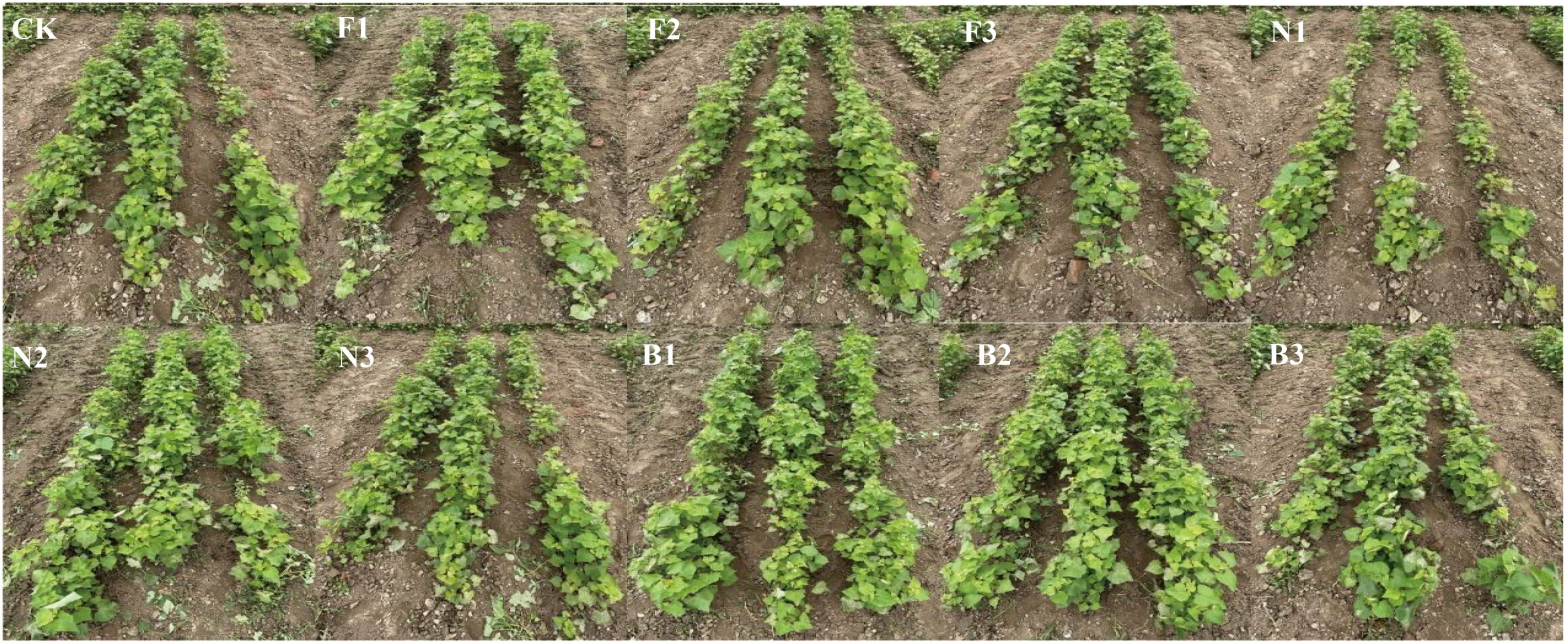
Field growth of buckwheat at seedling periods with different fertilization treatments CK: Fertilizer application rate of 0 kg·ha^-1^; F1: chemical fertilizer application rate of 225 kg·ha^-1^; F2: chemical fertilizer application rate of 300 kg·ha^-1^; F3: chemical fertilizer application rate of 375 kg·ha^-1^; N1: cow manure application rate of 7,500 kg·ha^-1^; N2: cow manure application rate of 15,000 kg·ha^-1^; N3: cow manure application rate of 22,500 kg·ha^-1^; B1: CBM fertilizer application rate of 7,500 kg·ha^-1^; B2: CBM fertilizer application rate of 15,000 kg·ha^-1^; and B3: CBM fertilizer application rate of 22,500 kg·ha^-1^.

The experiments used a randomized block design (RBD) with one factor, and each treatment was repeated three times. The seeds were sown on July 1, 2022, and July 5, 2023. Row sowing was used. The plot area was 6 m^2^ (5 m long, 1.2 m wide, three rows with 30 cm row spacing), and the planting density was 50 plants/m^2^. Observation lanes and isolation rows were established between each plot. The width of the observation lanes was 2 m, and the width of the isolation rows was 1 m to prevent the mixing of fertilizers. According to previous studies, 10 fertilization treatments were implemented: no fertilization (CK, 0 kg·ha^-1^), low concentration of chemical fertilizer (F1, 225 kg·ha^-1^), medium concentration of chemical fertilizer (F2, 300 kg·ha^-1^), high concentration of chemical fertilizer (F3, 375 kg·ha^-1^), low concentration of cow manure (N1, 7500 kg·ha^-1^), medium concentration of cow manure (N2, 15,000 kg·ha^-1^), high concentration of cow manure (N3, 22,500 kg·ha^-1^), low concentration of CBM biofertilizer (B1, 7500 kg·ha^-1^), medium concentration of CBM biofertilizer (B2, 15,000 kg·ha^-1^), and high concentration of CBM biofertilizer (B3, 22,500 kg·ha^-1^). The specific fertilization conditions are shown in **Table 1**. In each treatment, the fertilizer was applied to the plot as the base fertilizer once before planting, and no fertilizer was applied during the entire growth period. Thinning or filling of the seedlings was performed at the seedling stage to maintain the planting density at 50 plants/m^2^. The buckwheat grains in each plot were harvested when 70% of the buckwheat grains were mature (October 26, 2022, and October 28, 2023). During the flowering and grain-filling periods, artificial irrigation was performed according to the principle of no less than 80% of the field capacity, and natural precipitation was used in the other periods. The other field management and pest control methods used were the same as those used for local high-yield cultivation. The Tartary buckwheat growth status under the different fertilization treatments is shown in **Figure 1**. The average temperature, sunshine duration, and precipitation from July to October 2022 were 20.3°C, 269.4 h, and 34.5 mm, respectively, and those in 2023 were 22.7°C, 232.1 h, and 42.3 mm, respectively.

**Table 1:** Test treatment and fertilizer dosage.

Treatment	Rate of fertilizer application (kg ha^-1^)	N (kg ha^-1^)	P_2_O_5_ (kg ha^-1^)	K_2_O (kg ha^-1^)
CK	0	0	0	0
F1	225	33.75	16.88	40.50
F2	300	45.00	21.10	50.63
F3	375	56.25	26.50	63.29
N1	7500	22.5	11.25	27.00
N2	15000	45.00	22.50	54.00
N3	22500	67.50	33.75	81.00
B1	7500	22.50	10.50	24.83
B2	15000	45.00	21.00	49.65
B3	22500	67.50	31.50	74.48

CK: control without fertilization; F1: 225 kg ha^-1^; F2: 300 kg ha^-1^; F3: 375 kg ha^-1^; N1: 7–500 kg ha^-1^; N2: 15–000 kg ha^-1^; N3: 22–500 kg ha^-1^; B1: 7–500 kg ha^-1^; B2: 15–000 kg ha^-1^; B3: 22–500 kg ha^-1^.

### 2.2 Test fertilizer

Castor bean meal (CBM) biofertilizer (provided by the Key Laboratory of Castor Breeding State People’s Committee) had a pH value of 6.20, an organic carbon content of 69.00 g/kg, a total nitrogen content of 3.00 g/kg, a total phosphorus content of 1.40 g/kg, a total potassium content of 3.31 g/kg, an alkaline dissolved nitrogen content of 0.25 mg/kg, an alkaline phosphorus content of 2.70 mg/kg, and a fast-acting potassium content of 9.40 mg/kg. Castor bean meal (CBM) biofertilizer contained strains and enzyme activities are shown in **Table 2**. chemical fertilizer (purchased from Stanley Agricultural Group Co., Ltd.) total nutrient content ≥45%, N-P_2_O_5_-K_2_O:15-15-15. cow manure (provided by Tongliao Agricultural and Animal Husbandry Scientific Research Institute, Inner Mongolia) pH 6.50-8.50, organic matter content 14.50%, total nitrogen content 0.20%-0.45%, total phosphorus content 0.15%-0.25%, alkaline phosphorus content 0.15%-0.25%. 0.15%~0.25%, total potassium content was 0.10%~0.15%.

**Table 2:** Beneficial bacteria and enzyme activity of castor bean meal biofertilizer.

Index	Castor bean meal biofertilizer							
Strains	*Bacillus subtilis*	*Saccharomyces cerevisiae*	*Arthrospira platensis*	*Acetobacter aceti*	*Lactobacillus acidophilus*	*Streptomyces coelicolor*	*Aspergillus oryzae*	
Enzyme activity	Urease	Phosphatase	Sucrase	Catalase	Acid converting enzyme	Olyphenol oxidase	Protease	Cellulase

### 2.3 Sample preparation

During the seedling stage, flowering stage, grain-filling stage, and maturity stage, 30 Tartary buckwheat plants with stable growth and an intact root system were randomly excavated from each treatment plot. After rinsing with tap water, 20 Tartary buckwheat plants were selected for each treatment to measure the plant height, stem diameter, number of main stem nodes, and number of main stem branches. The leaves of the remaining 10 Tartary buckwheat plants (located at nodes 4–6 at the top of the main stem) were selected, One part was treated with liquid nitrogen for 30 s and then stored in a refrigerator at -80°C for the determination of chlorophyll content, and the other part was cleaned and stored in a refrigerator at 4°C for the determination of leaf blade area.

The specific mechanism of buckwheat sprout cultivation was as follows: 300 buckwheat grains were selected from each fertilization treatment, the seeds were washed three times with deionized water and then soaked for 5 min, the soaked seeds were placed into a disinfection tray and soaked in 1% NaClO solution for 15 min for disinfection, and the disinfected seeds were then washed three times and placed at room temperature (25°C). Tartary buckwheat seeds subjected to the different treatments were then soaked in sterile water for 24 h prior to germination. Incubation was performed for 16 d with 24 h as the base. At 0 d, 4 d, 8 d, 12 d, and 16 d of buckwheat sprout growth, 30 sprouts with consistent growth were selected, treated with liquid nitrogen for 30 s, and then stored in a -80°C freezer for the subsequent measurement of amino acid, soluble sugar, vitamin C, bioflavonoid, and total phenolic contents.

### 2.4 Measurements

In accordance with the methods of Yang et al. (2024), the plant height, stem diameter, number of main stem nodes, number of main stem branches, the grain weight per plant, and 1,000-grain weight were measured for each treatment during each growth period. An area of 1 m^2^ was randomly selected in the center of each plot (no sampling was performed, and the border plants were not included) for the collection of grains from all plants, and the grains were air-dried to measure the yield. The leaf blade area during each period was measured via a Handheld Laser leaf blade area Meter (CI-203, CID, USA) (Yuan et al., 2024a). The chlorophyll content was determined via a chlorophyll content kit (BC0995), which was purchased from Beijing Solarbio Science and Technology Co., Ltd.

The length, diameter, tap root length, fresh weight, and dry weight of the buckwheat sprouts obtained from each fertilization treatment were measured according to the methods of Starič et al. (2023). Three days after germination, the germination rates of Tartary buckwheat harvested under different fertilization treatments were measured with the sprout length of 2 mm as the base.

The buckwheat protein content was determined using the Protein Content Assay Kit (A045-2-2; Nanjing Jiancheng Bioengineering Institute, which utilizes the Coomassie brilliant blue G-250 method to determine protein content. The content of free amino acids in the buckwheat sprouts was determined using a free amino acid content detection kit (BC1575; Beijing Solarbio Science and Technology Co., Ltd. The anthrone method was used to determine the starch content. Briefly, 0.1 g of sample was weighed, 50 mL of 80% ethanol solution was added, and the solution was heated in a constant-temperature water bath at 45°C for 10 min. After suction filtration, 1 mL of the filtrate was added to a colorimetric tube, followed by the addition of 0.5 mL of anthrone solution and 4.5 mL of concentrated sulfuric acid. After shaking, the tube was covered and placed in a boiling water bath for 10 min. After cooling to room temperature, the optical density (OD) was measured at 620 nm use Tecan Spark multifunctional enzyme marker assay (Yang et al., 2019). The cellulose content was determined using a cellulose analyzer (SQ-XW06, Hebei Yunpu Analytical Instrument Co., Ltd.). A plant flavonoid content detection kit (BC1330; Beijing Solarbio Science and Technology Co., Ltd. was used to determine the plant flavonoid content in Tartary buckwheat and buckwheat sprouts; this kit uses the spectrophotometric method to determine the plant flavonoid content. The soluble sugar content in the sprouts was determined using a soluble sugar content detection kit (BC0030; Beijing Solarbio Science and Technology Co., Ltd. this kit uses the anthrone method. The total phenolic content in buckwheat sprouts was determined using a total plant phenolic content detection kit (BC1340; Beijing Solarbio Science and Technology Co., Ltd. and this kit uses the spectrophotometric method. The vitamin C content in the sprouts was determined using a vitamin C content detection kit (BC1234; Beijing Solarbio Science and Technology Co., Ltd. The bioflavonoid components were measured at ProNet Biotech Co., Ltd. using high-performance liquid chromatography-mass spectrometry(Tsushima LCMS-2050 High Performance Liquid Chromatography Mass Spectrometer). The fat content of Tartary buckwheat was determined by Soxhlet extraction method according to Lo Presti et al. (2023) Briefly, 2–5 g of dried and ground sample was accurately weighed, mixed with sea sand, and transferred to a filter paper tube. Then, the filter paper tube was placed into a Soxhlet extractor, anhydrous ether was added at the upper end of condenser, and the extractor was heated on a water bath to continuously reflux the ether for 6–12 h of extraction. After suction filtration, the receiving bottle was removed. When the bottle contained 1–2 cm of ether, the extractor was placed on a water bath to evaporate the ether and then dried at 100-105°C for 2 h. The extract was then removed and placed in a desiccator to cool for 30 min and weighed, and the process was repeated until a constant weight was obtained. The fat content was calculated according to the following formula:


Fat content=(M2−M1)/M×100%

where M_2_ is the sum of the masses of the receiving bottle and the fat, M1 is the mass of the receiving bottle, and M is the mass of the sample.

### 2.5 Statistical analysis

Microsoft Excel 2016 and SPSS 25.0 were used for data processing. All the data were analyzed via one-way analysis of variance (ANOVA) and multiple comparison test (Tukey). The differences among the treatments were compared (*P<*0.05). The growth rate under each fertilizer treatment was calculated using the formula (actual value - control value)/control value * 100%, and the rate of decrease was determined using the formula (control value - actual value)/control value * 100%. The results in 2022 and 2023 were similar. Therefore, the two-year averages are presented, and the data from each of these two years are presented as supplementary data. GraphPad Prism 10 was used to plot the data results and Photoshop 2020 was used for image processing.

## 3 Results

### 3.1 Effects of CBM biofertilizer on the agronomic traits of Tartary buckwheat

**Figure 2:**
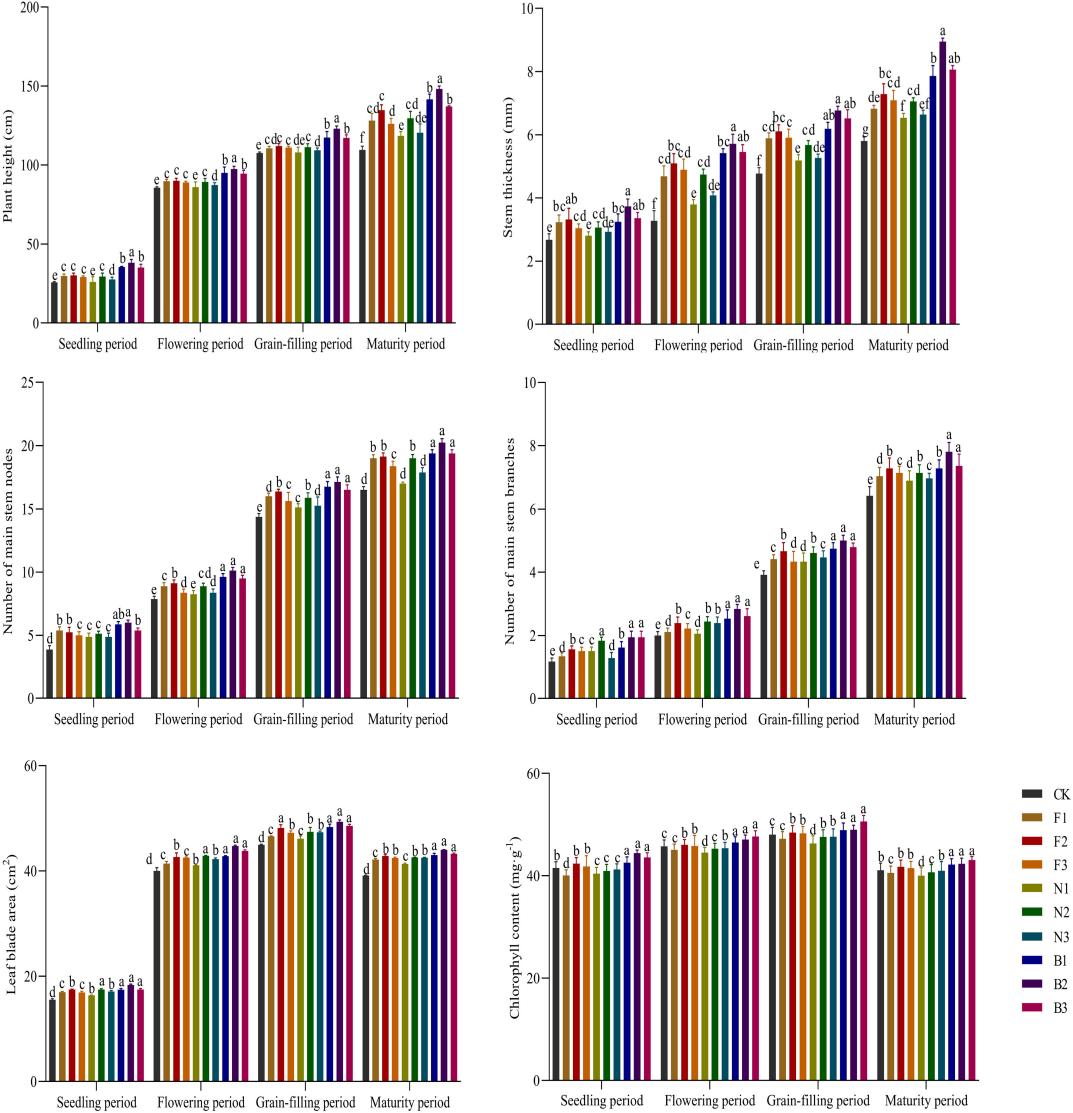
Effects of CBM biofertilizer on the agronomic traits of Tartary buckwheat Small letter in the same column means significant difference at *p*< 0.05. CK: Fertilizer application rate of 0 kg·ha^-1^; F1: chemical fertilizer application rate of 225 kg·ha^-1^; F2: chemical fertilizer application rate of 300 kg·ha^-1^; F3: chemical fertilizer application rate of 375 kg·ha^-1^; N1: cow manure application rate of 7,500 kg·ha^-1^; N2: cow manure application rate of 15,000 kg·ha^-1^; N3: cow manure application rate of 22,500 kg·ha^-1^; B1: CBM fertilizer application rate of 7,500 kg·ha^-1^; B2: CBM fertilizer application rate of 15,000 kg·ha^-1^; and B3: CBM fertilizer application rate of 22,500 kg·ha^-1^.

During the growth period, the plant height, stem diameter, number of main stem nodes, and number of main stem branches of Tartary buckwheat in the different fertilization treatments increased. The plant height, stem diameter, number of main stem nodes, and number of main stem branches under the CBM biofertilizer treatments (B1, B2, and B3) were greater than those under the no fertilizer, chemical fertilizer, and cow manure treatments, and there were significant differences between the B2 treatment and the other fertilization treatments (**Figure 2**). The leaf area and chlorophyll content of Tartary buckwheat first increased but then decreased as growth progressed, reaching a maximum at the grain-filling stage, and those in the B2 treatment were significantly greater than those in the other fertilization treatments. The plant height, stem diameter, number of main stem nodes, number of main stem branches, leaf blade area, and chlorophyll content increased first but then decreased with increasing fertilizer application rate, with the highest values under the B2 treatment and the lowest under the CK treatment. Compared with those in the CK, F1, F2, F3, N1, N2, N3, B1, and B3 treatments, the plant height in the B2 treatment on average was 23.81%, 13.52%, 10.85%, 14.50%, 20.19%, 13.11%, 18.02%, 4.42%, and 5.96% greater, respectively; the average stem diameter in the B2 treatment was 52.25%, 21.98%, 15.38%, 20.21%, 37.26%, 22.55%, 32.98%, 10.81%, and 7.54% greater, respectively; the average number of main stem nodes under the B2 treatment was 25.51%, 8.63%, 7.27%, 12.93%, 18.23%, 9.46%, 15.36%, 3.63%, and 5.42% greater, respectively; the number of main stem branches under the B2 treatment was on average 30.25%, 18.05%, 10.67%, 15.72%, 18.94%, 9.74%, 16.40%, 8.80%, and 5.21% greater, respectively; and the leaf blade area in the B2 treatment was on average 12.01%, 6.34%, 3.52%, 4.90%, 7.94%, 4.04%, 4.83%, 3.22%, and 2.19% greater, respectively.

### 3.2 Effects of the CBM biofertilizer on the yield of Tartary buckwheat

**Figure 3:**
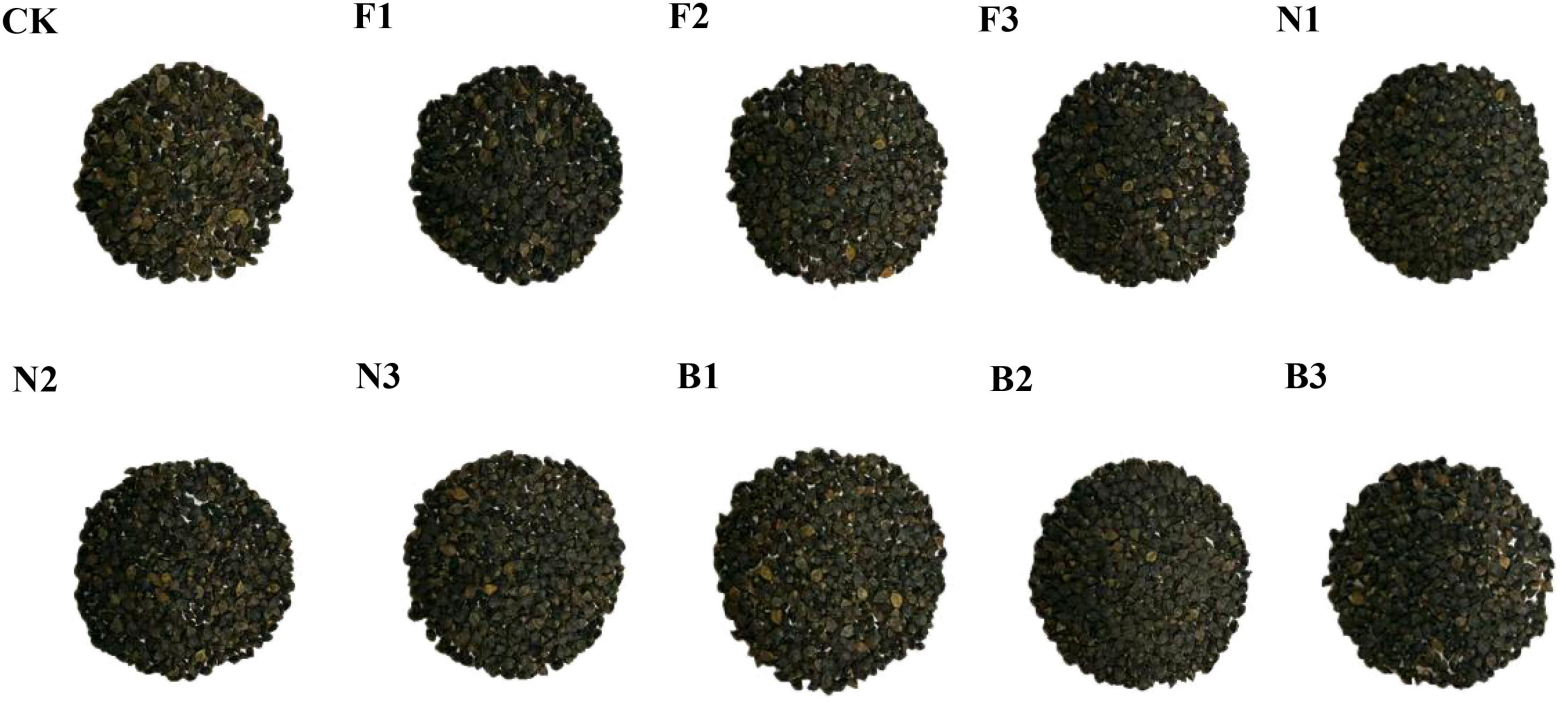
Buckwheat kernels harvested from different fertilization treatments CK: Fertilizer application rate of 0 kg·ha^-1^; F1: chemical fertilizer application rate of 225 kg·ha^-1^; F2: chemical fertilizer application rate of 300 kg·ha^-1^; F3: chemical fertilizer application rate of 375 kg·ha^-1^; N1: cow manure application rate of 7,500 kg·ha^-1^; N2: cow manure application rate of 15,000 kg·ha^-1^; N3: cow manure application rate of 22,500 kg·ha^-1^; B1: CBM fertilizer application rate of 7,500 kg·ha^-1^; B2: CBM fertilizer application rate of 15,000 kg·ha^-1^; and B3: CBM fertilizer application rate of 22,500 kg·ha^-1^.

**Figure 4:**
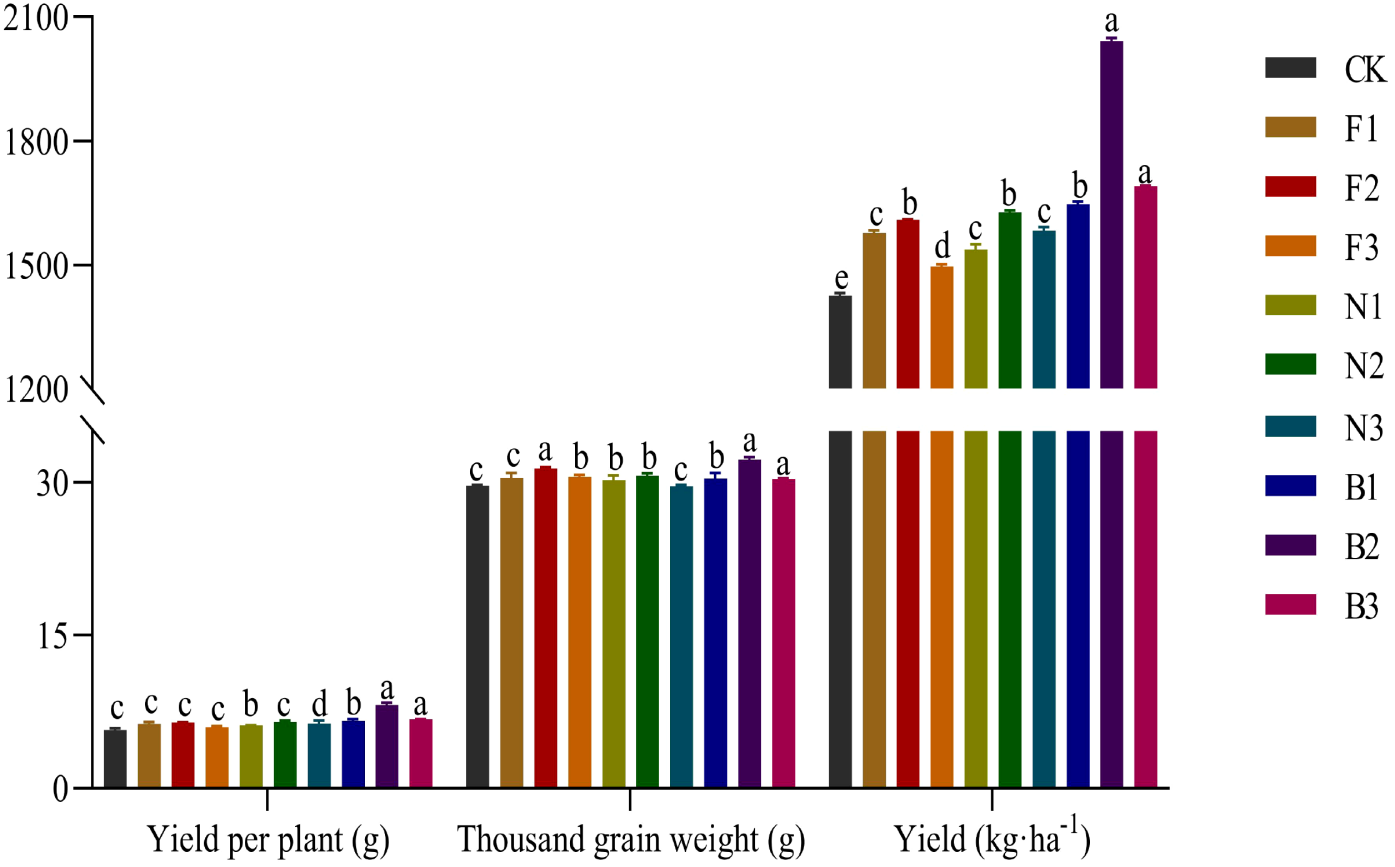
Effects of the CBM biofertilizer on the yield of Tartary buckwheat. Small letter in the same column means significant difference at *p*< 0.05. CK: Fertilizer application rate of 0 kg·ha^-1^; F1: chemical fertilizer application rate of 225 kg·ha^-1^; F2: chemical fertilizer application rate of 300 kg·ha^-1^; F3: chemical fertilizer application rate of 375 kg·ha^-1^; N1: cow manure application rate of 7,500 kg·ha^-1^; N2: cow manure application rate of 15,000 kg·ha^-1^; N3: cow manure application rate of 22,500 kg·ha^-1^; B1: CBM fertilizer application rate of 7,500 kg·ha^-1^; B2: CBM fertilizer application rate of 15,000 kg·ha^-1^; and B3: CBM fertilizer application rate of 22,500 kg·ha^-1^.

The different fertilization treatments significantly affected the grain weight per plant and the 1000-grain weight. **Figure 3** shows that the buckwheat grains harvested in the B2 treatment were relatively large and had a high fullness. With increasing fertilizer application rate, the grain weight per plant, the 1,000-grain weight, and the yield of Tartary buckwheat first increased but then decreased, with those of the B2 treatment being significantly greater than those of the other fertilization treatments. Compared with those in the CK, F1, F2, F3, N1, N2, N3, B1, and B3 treatments, the grain weight per plant in the B2 treatment was 43.12%, 29.40%, 26.79%, 36.31%, 32.66%, 25.33%, 27.98%, 23.81%, and 20.78% greater, respectively; the 1000-grain weight in the B2 treatment was 8.61%, 5.94%, 2.91%, 5.52%, 6.60%, 5.15%, 8.82%, 6.11%, and 6.29% greater, respectively; and the yield in the B2 treatment was 43.0%, 29.39%, 26.78%, 36.34%, 32.70%, 25.37%, 28.89%, 23.87%, and 20.74% greater, respectively (**Figure 4**).

### 3.3 Effects of CBM biofertilizer on the flavonoid components of Tartary buckwheat

**Figure 5:**
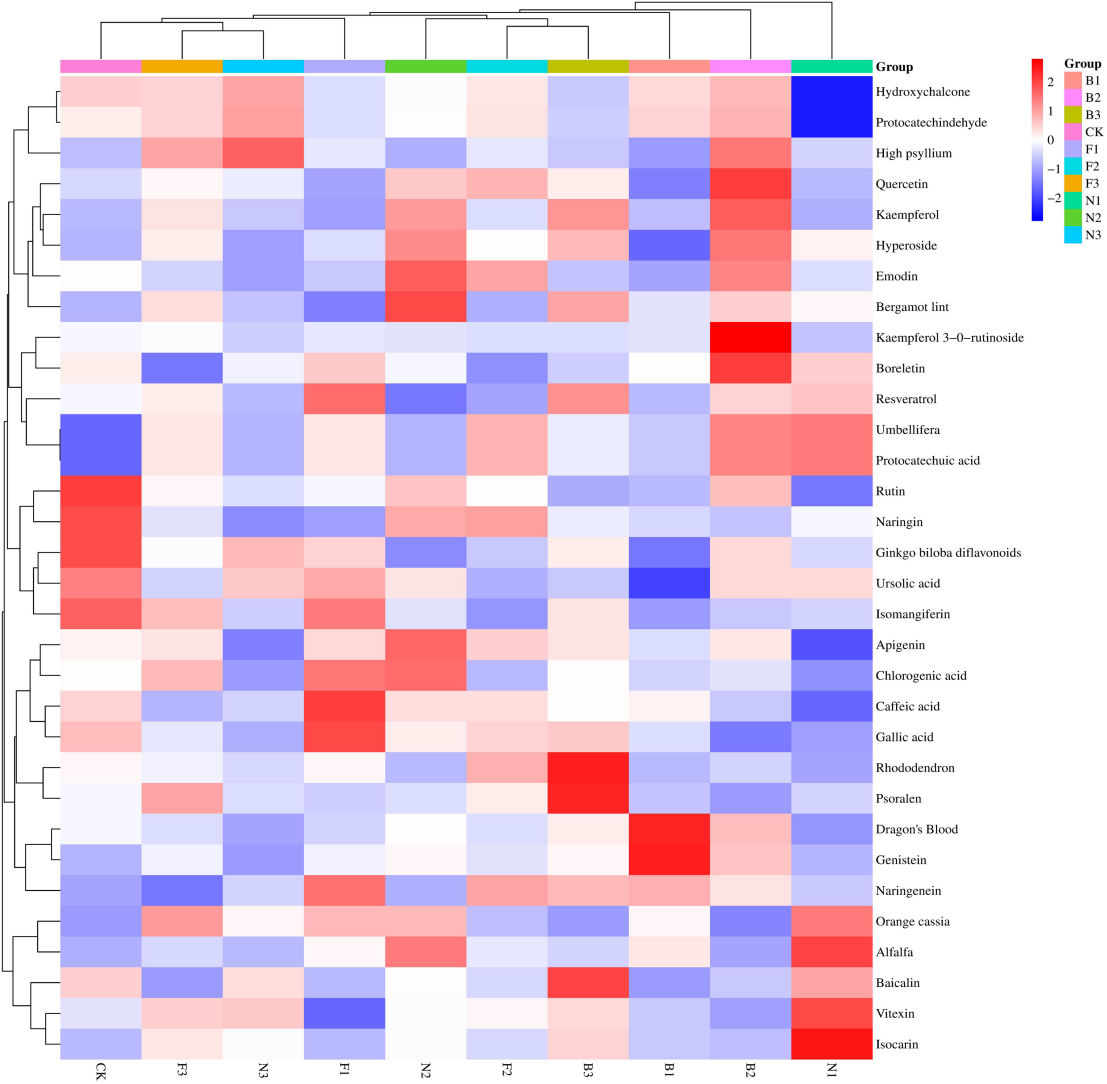
Buckwheat bioflavonoid fraction content clustering heat map Small letter in the same column means significant difference at *p*< 0.05. The abscissa is different fertilization treatments, and the vertical axis is the content of each component of flavonoids. Red represents high content and blue represents low content. CK: Fertilizer application rate of 0 kg·ha^-1^; F1: chemical fertilizer application rate of 225 kg·ha^-1^; F2: chemical fertilizer application rate of 300 kg·ha^-1^; F3: chemical fertilizer application rate of 375 kg·ha^-1^; N1: cow manure application rate of 7,500 kg·ha^-1^; N2: cow manure application rate of 15,000 kg·ha^-1^; N3: cow manure application rate of 22,500 kg·ha^-1^; B1: CBM fertilizer application rate of 7,500 kg·ha^-1^; B2: CBM fertilizer application rate of 15,000 kg·ha^-1^; and B3: CBM fertilizer application rate of 22,500 kg·ha^-1^.

The bioflavonoid components of the Tartary buckwheat grains harvested from the different fertilization treatments were determined, and 33 components were found, including quercetin, rutin, hyperoside, kaempferol, kaempferol 3-0-rutinoside, psoralen, bergamot lint, hydroxychalcone, dragon’s blood, alfalfa, boreletin, isomangiferin, genistein, baicalin, *Ginkgo biloba* diflavonoids, ruthenium naringenin, protocatechindehyde, protocatechuic acid, umbellifera, gallic acid, caffeic acid, resveratrol, apigenin, emodin, naringenin, rhododendron, high psyllium, orange cassia, chlorogenic acid, vitexin, isocarin, ursolic acid, and naringin. Different fertilization treatments had significant effects on these 33 flavonoids. Among them, the most abundant components were quercetin, rutin, hyperoside, kaempferol, and kaempferol 3-0-rutinoside, which have decisive effects on bioflavonoid synthesis (**Figure 5**). In terms of the effects of the different fertilization treatments on the contents of bioflavonoids, the contents of nine bioflavonoid components, namely, quercetin, protocatechuic acid, hyperoside, kaempferol, kaempferol 3-0-rutinoside, hydroxychalcone, boreletin, protocatechindehyde, and umbellifera, were greater under the B2 treatment than under the other treatments. For Tartary buckwheat in the CK treatment, the levels of five bioflavonoids, namely, rutin, isomangiferin, Ginkgo biloba diflavonoids, ruthenium naringenin, and naringin, were relatively high. In the N1 treatment, the levels of four bioflavonoids, including alfalfa, protocatechuic acid, vitexin, and isocarin, were relatively high. In the N2 treatment, the levels of four bioflavonoids, namely, emodin, bergamot lint, apigenin, and chlorogenic acid, were relatively high. In the B3 treatment, the levels of three bioflavonoids, namely, psoralen, baicalin, and chlorogenic acid, were relatively high. In the F1 treatment, the levels of three bioflavonoids, namely, gallic acid, caffeic acid, and resveratrol, were relatively high. In the B1 treatment, the levels of two bioflavonoids, namely, dragon’s blood and genistein, were relatively high. Compared with the other fertilization treatments, the B2 treatment had the greatest effect on the bioflavonoid components of Tartary buckwheat.

### 3.4 Effects of CBM biofertilizer on the quality of Tartary buckwheat

**Figure 6:**
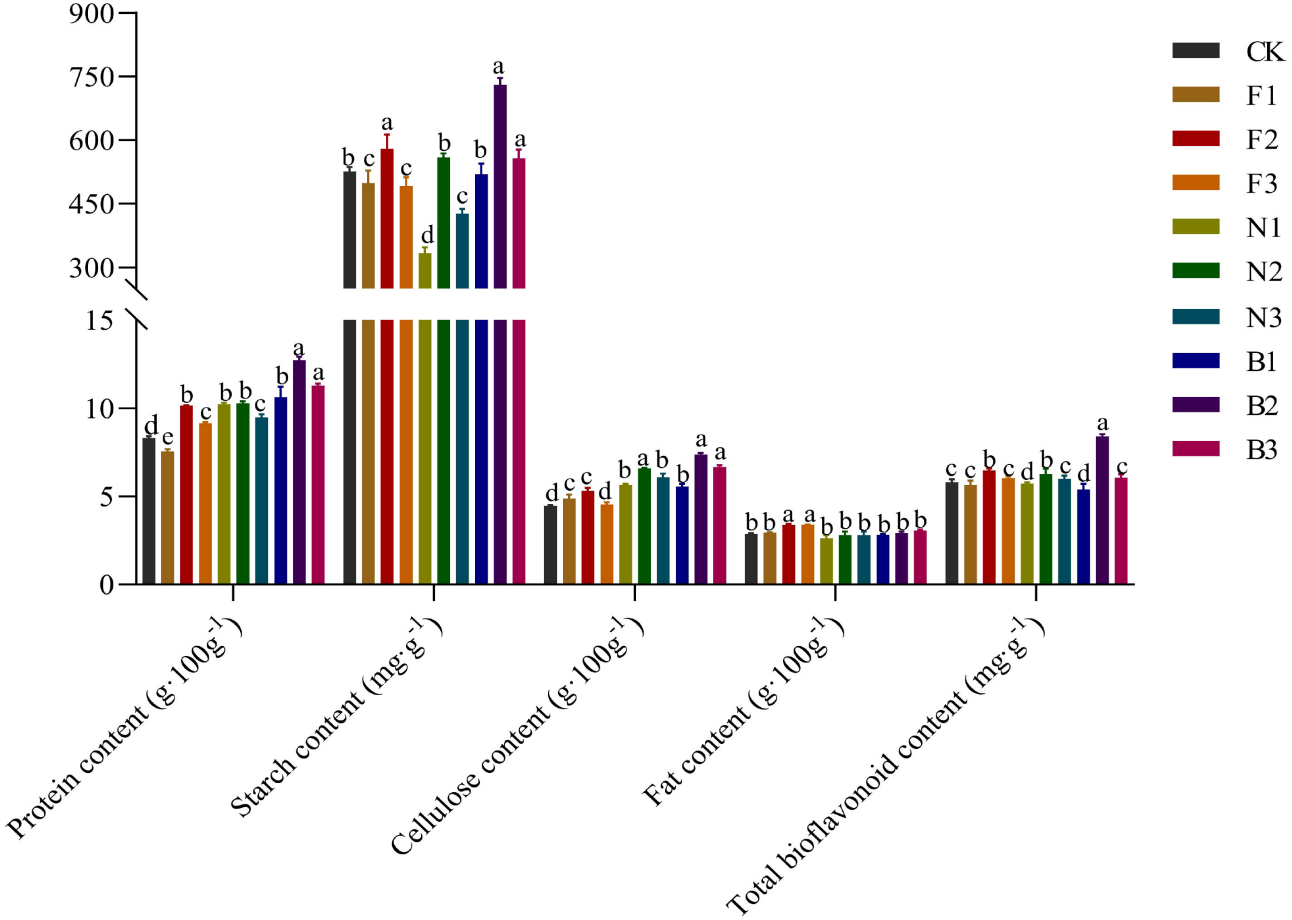
Effects of CBM biofertilizer on the quality of Tartary buckwheat. Small letter in the same column means significant difference at *p*< 0.05. CK: Fertilizer application rate of 0 kg·ha^-1^; F1: chemical fertilizer application rate of 225 kg·ha^-1^; F2: chemical fertilizer application rate of 300 kg·ha^-1^; F3: chemical fertilizer application rate of 375 kg·ha^-1^; N1: cow manure application rate of 7,500 kg·ha^-1^; N2: cow manure application rate of 15,000 kg·ha^-1^; N3: cow manure application rate of 22,500 kg·ha^-1^; B1: CBM fertilizer application rate of 7,500 kg·ha^-1^; B2: CBM fertilizer application rate of 15,000 kg·ha^-1^; and B3: CBM fertilizer application rate of 22,500 kg·ha^-1^.

With increasing fertilization application rate, the Tartary buckwheat protein, starch, cellulose, fat, and bioflavonoid contents first increased but then decreased, with the protein, starch, cellulose, and bioflavonoid contents in the B2 treatment being significantly greater than those in the other treatments. Among the fertilization treatments, the fat content in the F2 treatment was greater than that in the other fertilization treatments, but the differences among the treatments were relatively small (**Figure 6**). Compared with the CK, F1, F2, F3, N1, N2, N3, B1, and B3 treatments, the buckwheat protein content under the B2 treatment was 53.31%, 68.52%, 25.52%, 39.23%, 24.54%, 23.93%, 34.25%, 19.74%, and 12.94% greater, respectively; the starch contents in the B2 treatment were 38.74%, 46.34%, 25.80%, 48.60%, 119.12%, 30.65%, 71.27%, 40.64%, and 30.97% greater, respectively; the cellulose content under the B2 treatment was 65.32%, 51.12%, 38.65%, 62.42%, 30.80%, 12.14%, 21.35%, 33.15%, and 10.79% greater, respectively; and the bioflavonoid content in the B2 treatment was 45.0%, 48.85%, 29.98%, 39.47%, 47.29%, 27.62%, 38.10%, 51.53%, and 26.09% greater, respectively.

### 3.5 Effects of CBM biofertilizer on the yield of Tartary buckwheat sprouts

**Figure 7:**
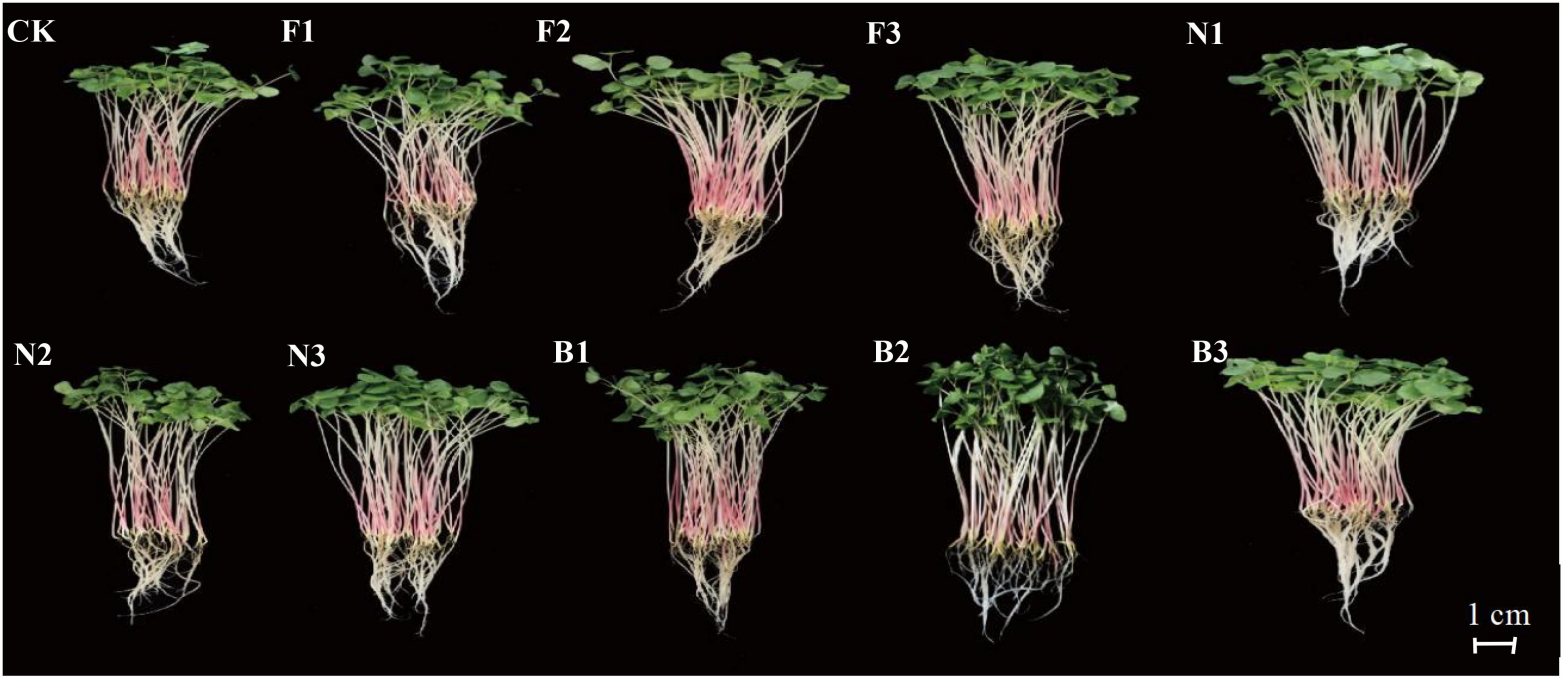
Growth of buckwheat buds in different fertilization treatments CK: Fertilizer application rate of 0 kg·ha^-1^; F1: chemical fertilizer application rate of 225 kg·ha^-1^; F2: chemical fertilizer application rate of 300 kg·ha^-1^; F3: chemical fertilizer application rate of 375 kg·ha^-1^; N1: cow manure application rate of 7,500 kg·ha^-1^; N2: cow manure application rate of 15,000 kg·ha^-1^; N3: cow manure application rate of 22,500 kg·ha^-1^; B1: CBM fertilizer application rate of 7,500 kg·ha^-1^; B2: CBM fertilizer application rate of 15,000 kg·ha^-1^; and B3: CBM fertilizer application rate of 22,500 kg·ha^-1^.

**Figure 8:**
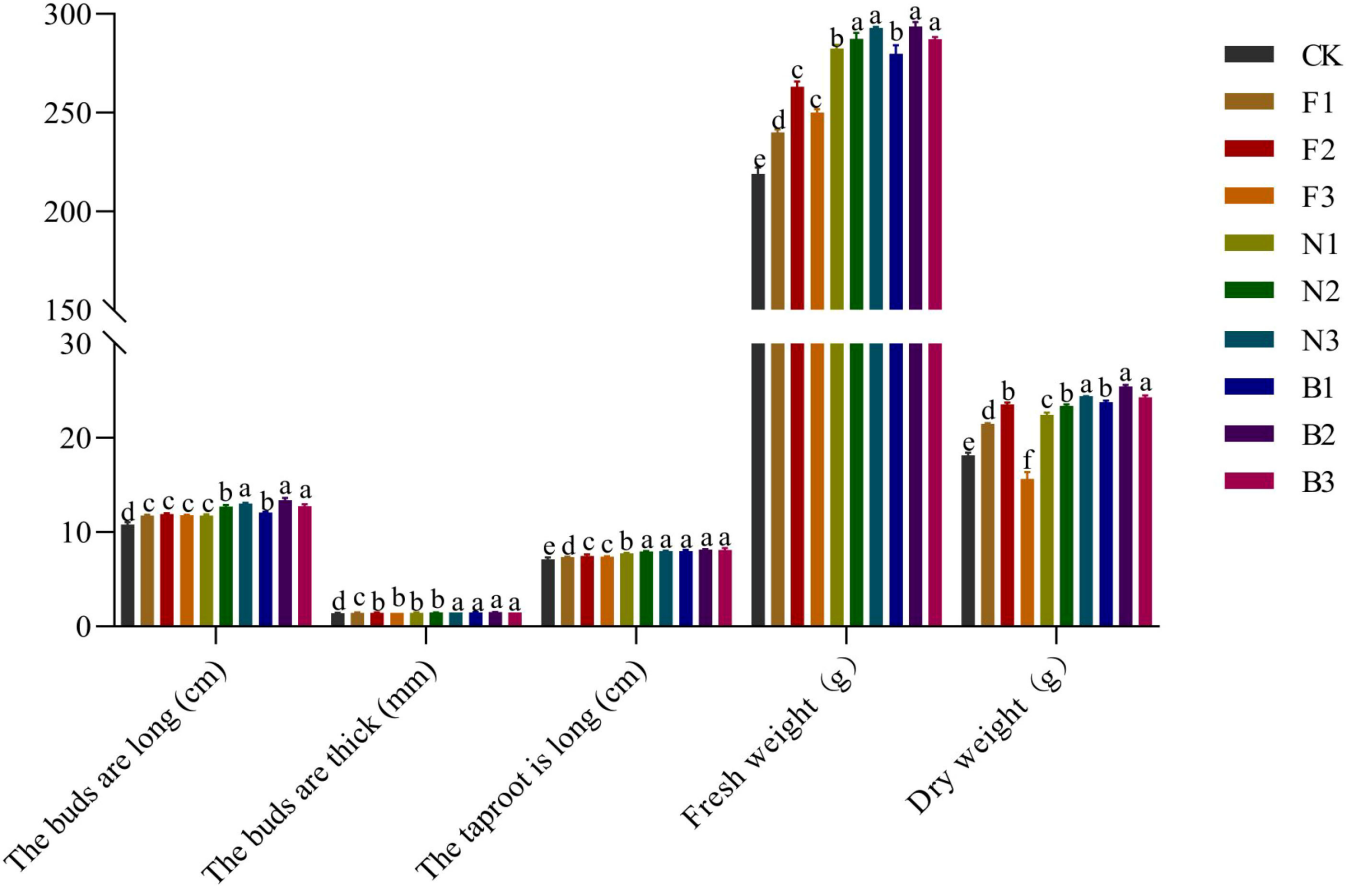
Effects of CBM biofertilizer on the yield of Tartary buckwheat sprouts. Small letter in the same column means significant difference at *p*< 0.05. CK: Fertilizer application rate of 0 kg·ha^-1^; F1: chemical fertilizer application rate of 225 kg·ha^-1^; F2: chemical fertilizer application rate of 300 kg·ha^-1^; F3: chemical fertilizer application rate of 375 kg·ha^-1^; N1: cow manure application rate of 7,500 kg·ha^-1^; N2: cow manure application rate of 15,000 kg·ha^-1^; N3: cow manure application rate of 22,500 kg·ha^-1^; B1: CBM fertilizer application rate of 7,500 kg·ha^-1^; B2: CBM fertilizer application rate of 15,000 kg·ha^-1^; and B3: CBM fertilizer application rate of 22,500 kg·ha^-1^.

There were significant differences in the sprout length, fresh weight, and dry weight of the Tartary buckwheat sprouts harvested from the different fertilization treatments (**Figure 7**). With increasing fertilizer application rate, the sprout length, sprout diameter, tap root length, fresh weight, and dry weight of the sprouts from each treatment first increased but then decreased. The sprout length, fresh weight, and dry weight of the sprouts were significantly greater under the B2 treatment than under the other fertilization treatments. Compared with the CK, F1, F2, F3, N1, N2, N3, B1, and B3 treatments, the length of the sprouts cultured from buckwheat harvested in the B2 treatment was 24.17%, 13.84%, 12.59%, 13.55%, 13.84%, 5.51%, 2.92%, 10.83%, and 5.01% greater, respectively; the fresh weight was 34.13%, 22.38%, 11.61%, 17.39%, 3.93%, 2.16%, 0.21%, and 4.91% greater, respectively; and the dry weights were 40.07%, 18.54%, 7.98%, 62.62%, 13.46%, 8.81%, 4.26%, 7.02%, and 4.78% higher, respectively. The sprout diameters and tap root lengths of the sprouts bred from buckwheat harvested under the different fertilization treatments were similar, with small differences among the treatments (**Figure 8**).

### 3.6 Effects of CBM biofertilizer on the quality of Tartary buckwheat sprouts

**Figure 9:**
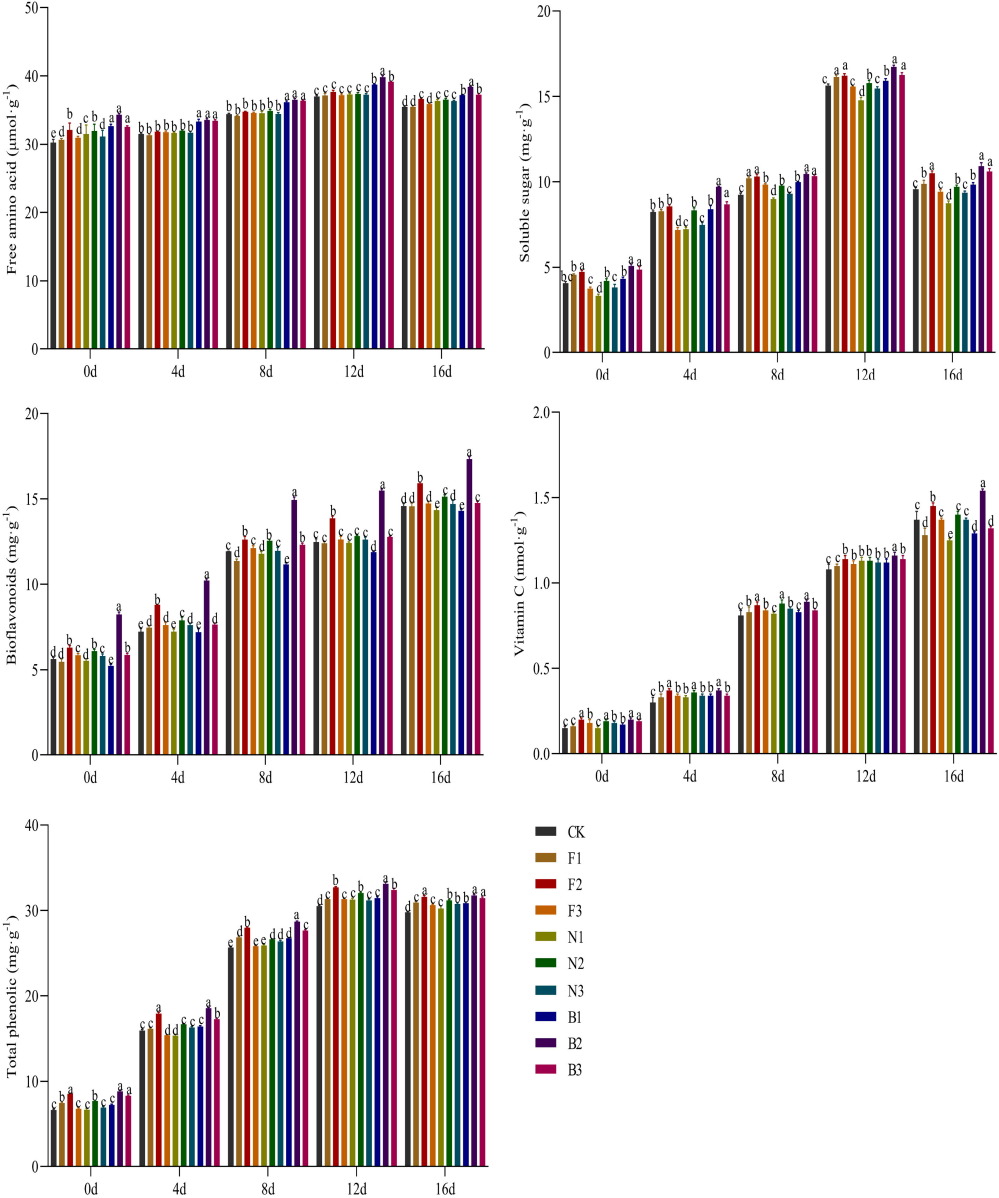
Effects of CBM biofertilizer on the quality of Tartary buckwheat sprouts. Small letter in the same column means significant difference at *p*< 0.05. CK: Fertilizer application rate of 0 kg·ha^-1^; F1: chemical fertilizer application rate of 225 kg·ha^-1^; F2: chemical fertilizer application rate of 300 kg·ha^-1^; F3: chemical fertilizer application rate of 375 kg·ha^-1^; N1: cow manure application rate of 7,500 kg·ha^-1^; N2: cow manure application rate of 15,000 kg·ha^-1^; N3: cow manure application rate of 22,500 kg·ha^-1^; B1: CBM fertilizer application rate of 7,500 kg·ha^-1^; B2: CBM fertilizer application rate of 15,000 kg·ha^-1^; and B3: CBM fertilizer application rate of 22,500 kg·ha^-1^.

With increasing duration of cultivation, the contents of free amino acids, soluble sugars, and total phenolics in the Tartary buckwheat sprouts under the different fertilization treatments first increased but then decreased, with the highest contents occurring at 12 d of cultivation(**Figure 9**); for the buckwheat harvested under the B2 treatment, the contents of free amino acids, soluble sugars, and total phenolics of the cultured sprouts were significantly greater than those under the other fertilization treatments. With increasing cultivation period, the vitamin C and bioflavonoid contents of the sprouts under the different fertilization treatments continuously increased, with the vitamin C and bioflavonoid contents of the sprouts cultivated from buckwheat harvested under the B2 treatment being significantly greater than those under the other fertilization treatments. The contents of free amino acids, soluble sugars, vitamin C, bioflavonoids, and total phenolics in the sprouts bred from grains harvested under the different fertilization treatments were significantly different and initially increased and then decreased with increasing fertilizer application rate, with the highest values under the B2 treatment and the lowest under the CK treatment. Compared with the CK, F1, F2, F3, N1, N2, N3, B1, and B3 treatments, the free amino acid content of the sprouts cultured from buckwheat harvested in the B2 treatment was on average 8.27%, 8.21%, 5.59%, 7.20%, 6.56%, 5.76%, 6.84%, 2.57%, and 2.14% greater, respectively, the soluble sugar content was on average 13.13%, 7.78%, 5.13%, 15.60%, 22.77%, 10.67%, 16.42%, 9.14%, and 4.22% greater, respectively, the vitamin C content was on average 12.13%, 12.43%, 3.23%, 8.33%, 13.04%, 5.05%, 7.77%, 10.93%, and 8.62% greater, respectively, the bioflavonoid contents were on average 27.70%, 29.14%, 15.15%, 25.02%, 29.04%, 21.49%, 25.59%, 32.93%, and 24.08% greater, respectively, and the total phenolic content was on average 11.38%, 7.30%, 1.84%, 10.03%, 10.49%, 5.88%, 8.44%, 7.34%, and 3.31% greater, respectively.

## 4 Discussion

### 4.1 Effect of different fertilization treatments on the agronomic traits of Tartary buckwheat

The agronomic traits of Tartary buckwheat during the growth process are closely associated with the grain weight and final yield. Plant height is an important agronomic trait in Tartary buckwheat. Buckwheat plants that are too tall are potentially prone to lodging, and plants that are too short may exhibit poor nutrition; therefore, plant height may affect buckwheat yield to some extent (Jha et al., 2024). Many studies have shown that the plant height of Tartary buckwheat under different fertilization treatments varies during different periods (Fang et al., 2018). Zhou et al. (2023b) reported that, compared with CK, different organic fertilizer application rates increased the plant height and improved agronomic traits. Tang Z. et al. (2024) reported that appropriate organic fertilizer treatment increased the plant height. In this study, compared with no fertilization, chemical fertilizer, or cow manure, CBM biofertilizer significantly increased the plant height of Tartary buckwheat, with the B2 treatment resulting in the maximum plant height, consistent with previous results. This result may be due to the rich nitrogen, phosphorus, and potassium contained in the CBM fertilizer, thus meeting the plant’s needs for nitrogen, phosphorus, and potassium during different growth periods. A sufficient nutrient supply can significantly promote the growth of Tartary buckwheat plants, thus increasing their height (Tang L. et al., 2024). Zhang et al. (2021) applied organic fertilizer to corn and reported significant improvement in plant traits, increased stalk diameter, and significant promotion of plant growth. The results of this study were consistent with these results, possibly because the CBM biofertilizer contains not only the basic nutrients needed for Tartary buckwheat growth but also trace elements and organic nutrients, which provide a rich material base for growth and contribute to stem growth and development, thereby increasing stem diameter (Xiang et al., 2019). Yang et al. (2024) reported that biological fertilizer can significantly affect the number of main stem nodes and the number of main stem branches and is beneficial for the environmental adaptability of buckwheat, thus improving the yield. In this study, compared with no fertilization, chemical fertilizer, or cow manure, the application of CBM biofertilizer increased the number of main stem nodes, and consistent with previous results, the B2 treatment had the greatest effect among the treatments, possibly because the beneficial microorganisms in the CBM biofertilizer can produce growth-promoting substances, such as auxin, which can increase the number of main stem nodes and the number of main stem branches in Tartary buckwheat (Zheng et al., 2023). Rotili et al. (2023) revealed that the leaf blade area decreased during the maturity stage in buckwheat because the accumulated dry matter in the plant transferred to the grains, consistent with the leaf growth pattern during each growth period in this study. Chlorophyll participates in photosynthesis in plant leaves. Zhou et al. (2023a) reported that the application of nitrogen fertilizer and increasing the planting density increased the chlorophyll content in Tartary buckwheat leaves. In this study, compared with no fertilization, chemical fertilizer, or cow manure, CBM biofertilizer significantly increased the chlorophyll content of the leaves, and consistent with previous results, the B2 treatment had the highest chlorophyll content, possibly because the CBM biofertilizer can significantly increase the photosynthetic intensity, promote chlorophyll synthesis, increase the leaf blade area, and increase the accumulation of photosynthetic products (Hornyák et al., 2022).

### 4.2 Effect of different fertilization treatments on the yield of Tartary buckwheat

The grain weight per plant and the 1,000-grain weight are important factors in determining the Tartary buckwheat yield. The higher the grain weight per plant is, the greater the yield, whereas the 1,000-grain weight indirectly affects yield by affecting the grain weight per plant and grain quality (Sobhani et al., 2014). Popović et al. (2013) showed that compared with that under conventional planting, the grain weight per plant and 1,000-grain weight of buckwheat plants grown with organic fertilizer were significantly greater, 7.28% and 9% respectively, indicating that organic fertilizer can increase buckwheat yield. Radchenko et al. (2018) reported that the application of organic fertilizer in the planting of buckwheat significantly increased the grain weight per plant, the 1000-grain weight, and the yield in southern Ukraine. Guo et al. (2022) combined organic straw and inorganic fertilizer and reported that, compared with CK, the combined application of organic manure and organic fertilizer significantly increased the grain weight per plant, the 1000-grain weight, and the final yield of buckwheat through increases in the soil nutrient content and improvements in the soil physicochemical environment. In this study, the application of CBM biofertilizer significantly increased the grain weight per plant, the 1,000-grain weight, and the yield of Tartary buckwheat, with the B2 treatment resulting in the highest grain weight per plant, 1,000-grain weight, and yield, 1.43, 1.08, and 1.43 times those of CK, respectively, indicating that CBM biofertilizer significantly increased the yield. This finding is consistent with the results of previous studies, possibly because CBM biofertilizer contains a variety of bacteria and soil enzymes that not only improve the soil environment and promote growth and development buckwheat but also improve plant immunity and increase disease resistance, which ultimately manifests as a significant increase in yield. The appropriate application of CBM biofertilizer may be beneficial for the absorption of soil nutrients by Tartary buckwheat plants, can promote the growth and development of Tartary buckwheat plants, increase the plant height, stem diameter, number of main stem nodes, and number of main stem branches, improve the leaf photosynthetic rate, increase the leaf blade area and chlorophyll content, and thus increase the aboveground biomass and yield (Yuan et al., 2024b).

### 4.3 Effect of different fertilization treatments on the quality of Tartary buckwheat

The quality of crops directly affects their nutritional value and economic benefits. Some studies have shown that fertilization is conducive to the absorption of nutrients by crops and plays an important role in improving the nutritional quality of grains (El-Saadony et al., 2021). Gao et al. (2024) reported that, compared with no fertilization, the application of an appropriate amount of organic fertilizer significantly increased the starch, cellulose, and fat contents of buckwheat. Gao et al. (2023) reported that appropriate applications of nitrogen fertilizer and organic fertilizer can promote endosperm development and starch synthesis in buckwheat, thereby increasing the contents of starch, cellulose, fat, and bioflavonoids in buckwheat grains. Wan et al. (2021) reported that fertilization can promote buckwheat starch anabolism and amino acid biosynthesis and increase the starch and protein contents of buckwheat grains. In this study, compared with no fertilization, chemical fertilizer, or cow manure, CBM biofertilizer significantly increased the protein, starch, cellulose, fat, and bioflavonoid contents of Tartary buckwheat grains, with the B2 treatment having the highest contents. This finding was consistent with the results of previous studies, possibly because, on the one hand, CBM biofertilizer contains a variety of microorganisms, and application of CBM to soil can promote soil microbe activity, increase soil biodiversity, and promote buckwheat growth and development, thereby improving yield and quality (Martinez et al., 2022); on the other hand, CBM biofertilizer contains high amounts of nitrogen, phosphorus, potassium, and trace elements, and nitrogen, phosphorus, and potassium can be used to develop a buckwheat root system and improve plant photosynthesis, organic synthesis, and transport ability, thus improving yield and quality (Çürük et al., 2020). Habtemariam (2019) reported that the application of green manure can promote the synthesis of rutin, quercetin, kaempferol, and resveratrol in buckwheat, thereby improving the bioflavonoid content and antioxidant activity. Jiang et al. (2007) found that fertilization may significantly increase the contents of rutin, procyanidin, gallic acid, and naringenin in buckwheat grains. Lee et al. (2016) observed that fertilization can regulate the synthesis and metabolism of flavonoid components (such as rutin, quercetin, and catechin), thereby increasing the total flavonoid content. The results of this study revealed that the B2 treatment significantly increased the contents of nine flavonoid components, all of which are key substances for bioflavonoid synthesis; these results are consistent with previous results, suggesting that CBM biofertilizer promotes bioflavonoid synthesis, possibly because some components of CBM biofertilizer may directly or indirectly participate in physiological metabolic processes in buckwheat, affecting flavonoid component synthetic pathways and regulatory mechanisms and thereby increasing flavonoid content (Li et al., 2019).

### 4.4 Effect of different fertilization treatments on buckwheat sprouts

Buckwheat sprouts are buckwheat seeds germinated under the right conditions to form young shoots, rich in a variety of bioactive ingredients (Kim et al., 2004). Rauf et al. (2019) observed significant differences in the sprout length, sprout diameter, fresh weight, and dry weight of buckwheat sprouts obtained from buckwheat harvested under different fertilization treatments. In this study, compared with buckwheat sprouts obtained from buckwheat harvested under no fertilizer, chemical fertilizer, and cow manure treatments, the sprout length, sprout diameter, tap root length, fresh weight, and dry weight of sprouts harvested in the B2 treatment were the greatest, consistent with previous results. These findings indicate that CBM biofertilizer can indirectly affect the growth status of buckwheat sprouts by affecting the nutrient contents of buckwheat grains. Studies have shown that after breeding of buckwheat sprouts, the contents of free amino acids, soluble sugars, vitamins, flavonoids, and phenolic substances increase significantly (Sytar et al., 2018). Dong et al. (2023) reported that during cultivation, the contents of free amino acids, flavonoids, and phenolic substances constantly changed, presenting a greater nutritional value than that of buckwheat grains. Zhang et al. (2015) reported that germination has significant effects on the nutritional components and various bioactive components of buckwheat, and the differences between grains subjected to different treatments could be visually observed through buckwheat sprouts. The results of this study showed that, compared with the use of buckwheat sprouts obtained from plants under no fertilizer, chemical fertilizer, or cow manure treatment, the application of CBM biofertilizer improved the quality of Tartary buckwheat, thereby indirectly increasing the contents of free amino acids, soluble sugars, vitamin C, bioflavonoids, and total phenolics, and the effect of the B2 treatment was the most significant. This finding is consistent with the results of previous studies and may be due to the greater quality of Tartary buckwheat plants grown with CBM biofertilizer, which can provide sufficient nutrients for sprout growth and development. During germination, macromolecular proteins, polysaccharides, and other substances in buckwheat undergo moderate decomposition by a variety of enzymes and are converted into free amino acids and soluble sugars and other substances that are easily absorbed by the human body, thereby affecting the quality of buckwheat sprouts (Borgonovi et al., 2023).

Notably, with a further increase in the CBM biofertilizer application rate, the yield and quality of Tartary buckwheat decreased, possibly due to an excess of nutrients in the soil, such as nitrogen, phosphorus, and potassium, caused by the excessive application of CBM biofertilizer. These surplus nutrients may not be effectively used by buckwheat but may instead inhibit the uptake of other nutrients, affecting normal crop growth and development and resulting in reduced yield and quality (Lu and Tian, 2017). In addition, excessive CBM biofertilizer may generate acidic substances or harmful gases during decomposition, which may damage the crop root system and affect normal crop growth and development, eventually affecting yield and quality (Li et al., 2018). In general, CBM biofertilizer has a positive effect on the growth and development of Tartary buckwheat and Tartary buckwheat sprouts, but farmers should reasonably control the amount of fertilizer applied. These findings indicate that high Tartary buckwheat yield and quality can be achieved through CBM biofertilizer application and that CBM fertilizer should be used in the planting of Tartary buckwheat. However, this study has some limitations, in this study, different fertilization treatments in buckwheat bioflavonoid fractions have significant differences, but did not further explore the reasons for the differences in bioflavonoid fractions, therefore, in the future, different fertilization treatments can be carried out on the buckwheat genomics and metabolomics of the joint analysis of gene expression of individual genes in the synthesis of bioflavonoids, to verify whether the gene up-regulation or down-regulation whether it led to differences in bioflavonoid fractions between different fertilization treatments.

## 5 Conclusion

An appropriate amount of CBM biofertilizer (15,000 kg·ha^-1^) increased the plant height, stem diameter, number of main stem nodes, and number of main stem branches, increased the leaf blade area and chlorophyll content, and increased the grain weight per plant and the 1,000-grain weight, thereby increasing the yield of Tartary buckwheat. This amount of CBM biofertilizer increased the contents of protein, starch, cellulose, and fat in Tartary buckwheat grains and increased the bioflavonoid content by promoting the synthesis of flavonoids. For buckwheat plants grown under this amount of CBM biofertilizer, after the harvested Tartary buckwheat grains were bred into Tartary buckwheat sprouts, the sprout length, sprout diameter, tap root length, and fresh weight of the sprouts were improved, and the contents of free amino acids, soluble sugars, vitamin C, total phenols, and bioflavonoids were greater than those in the other treatments. In order to achieve high-yield and high-quality cultivation of buckwheat, it is recommended that the CBM biofertilizer as a new type of bio-organic fertilizers used to reduce the amount of fertilizers and increase the efficiency of the promotion of green and organic agriculture to provide theoretical guidance for sustainable development.

## Data Availability

The original contributions presented in the study are included in the article/supplementary material. Further inquiries can be directed to the corresponding authors.
